# Management of child with acute airway obstruction: a case report

**DOI:** 10.1186/1757-1626-2-7517

**Published:** 2009-05-18

**Authors:** Antigona Hasani, Hajdin Thaqi, Shefki Azizi

**Affiliations:** 1 University Clinical Centre of Kosova, Department of Anesthesiology and Intensive CareMother Theresa n.n., 10000 PrishtinaRepublic of Kosova; 2 University Clinical Centre of Kosova, Department of OtolaryngologyMother Theresa n.n., 10000 PrishtinaRepublic of Kosova

## Abstract

**Introduction:**

Laryngotracheobronchitis is a rare, but in severe cases may lead to laryngeal edema progressing to complete closure of the subglottis within a few hours, without prompt treatment.

**Case presentation:**

We present the case of acute airway obstruction caused by laryngotracheobronchitis in a 6-year-old boy, initially misdiagnosed and treated as foreign body aspiration. The treatment of airway obstruction in the child was managed with algorithm used in our institution.

**Conclusion:**

The aims of management of acute airway obstructions are firstly, to maintain and secure the airway and than, to establish a diagnosis. Institutions should have algorithms to manage children with acute airway obstruction.

## Introduction

Management of acute airway obstruction is a challenge for anesthesiologist, otolaryngologist and pediatrician. Acute airway obstructions in children have multiple etiologies [1,2]. Laryngotracheobronchitis (LTB) is an acute inflammatory condition of the subglottic portion of the trachea and tracheobronchial tree. Inflammatory edema and viscoid secretions may rapidly obstruct the upper airway tract, resulting in asphyxia, hypoxic brain damage or death without prompt treatment [3]. Viscous crusts were removed by bronchoscopy. Tracheotomy is not usually required [4,5].

LTB occurs in children from ages 1 to 6 years, and most frequently in male gender [6]. The initial infection is attributed to a virus, with the infected mucosa secondarily invaded by bacteria [7].

Foreign bodies that involve in the larynx associated with a high degree of obstruction and a high mortality [8]. We present the case of acute laryngotracheobronchitis in a 6-year-old boy initially misdiagnosed and treated as foreign body aspiration.

## Case presentation

A 6-year-old Albanian boy presented in emergency unit of our hospital with signs of acute airway obstruction and stridor. These signs and symptoms had begun the day before, with a grippal infection and had rapidly progressed. In history occurred that child ate peanuts the same day. On arrival, the boy was in the severe condition. He was restless and agitated, in the “chest-knee'” position, with dyspnea, tachypnea and audible inspiratory stridor in the head up position. Cyanosis was present on the fingernails and lips. The chest examination revealed bilaterally decreasing breath sounds, with transmitted sounds from the upper airway, wheezing and whispery sounds.

His vital signs upon admission were as follows: temperature, 37.8ºC; blood pressure, 90/60 mmHg; heart rate, 150-160 beats/min; respiratory rate, 30-35 breaths/min and peripheral oxygen saturation, 85-87%.

**Figure 1.**
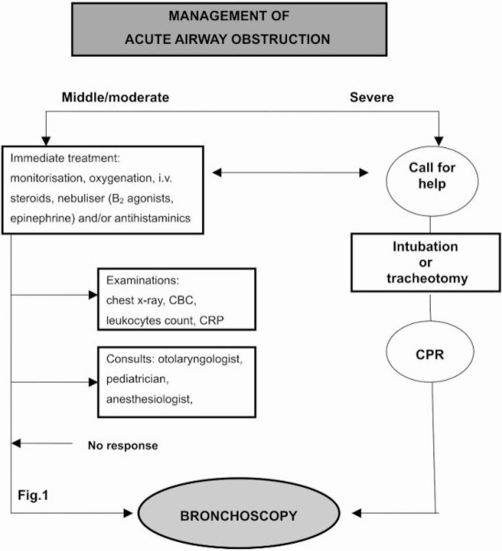
Algorithm for management of acute airway obstruction in University Clinical Centre of Kosova. CBC, complete blood count; CRP, C-reactive protein; CPR, cardiopulmonary resustation.

The treatment of airway obstruction in the child was managed with algorithm used in our institution (Figure 1).

**Figure 2.**
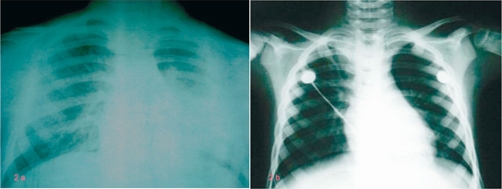
**(a)** Chest-X ray before bronchoscopy; **(b)** Chest-X ray after bronchoscopy.

Systemic corticosteroids (dexamethasone) and 100% oxygen via facial musk were performed immediately. After pediatrician, anesthesiologist and otolaryngologist consultations and chest-X ray (Figure 2a) the initially diagnosis at this stage was foreign body aspiration. The same day, the emergent direct bronchoscopy was performed.

The induction at anesthesia was made by combination of intravenous propofol (1 mg·kg^−1^) and inhalation of halothane (2-4%) in 100% O_2_. Anesthesia was maintained with continuous infusion of propofol, mean dose 8 mg kg h^1^ and 100% O_2_.

The otolaryngologist performed direct bronchoscopy with rigid bronchoscope. Oxygen was delivered through a side arm of the bronchoscope. Initial direct bronchoscopy showed bizarre anatomy, a tiny laryngeal inlet almost totally occluded by densely purulent secret, viscous crusts and edema in both bronchi. Crusts were removed by bronchoscopy and the secret was aspirate from airways. The surrounding tissue was sprayed with 3 ml of 4% lidocaine.

At the end of procedure, the child was intubated with uncuffed size 5.0 tracheal tube and transferred in the intensive care unit (Figure 2b presents chest radiography immediately after bronchoscopy).

In intensive care unit, the mechanical ventilation was sated in BIPAP mode and sedation with propofol 1 mg kg h^−1^ was continued. O_2_ saturation was 95%.

Antiedematous therapy was performed with dexamethasone 0.6 mg kg ^−1^. Bacterial and viral cultures were obtained at bronchoscopy and *Staphylococcus aureus* and nontypeable influenza virus was isolated. Antimicrobial therapy due to antibiogram was performed.

On day three, control examination with fiberoptic bronchoscope revealed significant regression of the edema and granulation tissue; endotracheal tube was removed and the patient woke up uneventfully with no further airway problems. The child was discharged from ICU in day 4.

## Discussion

Severe LTB is a rare but fatal infection and within a few hours, laryngeal edema can progress to complete closure of the subglottis. Misdiagnosis is common, with up to 30% of children treated as asthma, croup or foreign body aspiration [7]. Smaller objects can wedge in the larynx and cause inflammatory reactions, mimicking infective disease processes [9]. Our patient did have a history of eating peanuts the same day, despite this, his stridor and dyspnea were treated as a foreign body aspiration. However, the rapid onset of airway obstruction favored the diagnoses of foreign body aspiration. Only during bronchoscopy the exact diagnosis was lead.

In our case, we perform bronchoscopy under deep inhalational and intravenous anesthesia without the use of muscle relaxants. Paralysis by neuromuscular blockade may precipitate complete airway obstruction. The use of continuous positive airway pressure may help to maintain functional residual capacity that is reduced under anesthesia [10].

Placement of a tracheostomy tube in young patients is associated with a higher complication rate than in adults. Tracheostomies have a higher incidence of complications, such as bleeding, trauma to local structures, pneumothorax or pneumomediastinum [11]. The postoperative bleeds in this patient highlights the need for meticulous care in placement in small children. This reasons lead as to perform the second bronchoscopy in which the significant regression of the edema and granulation tissue occurred and endotracheal tube was removed without a need to tracheostomy.

In our institution, we performed algorithm for the evaluation and treatment of children with airway obstruction. The algorithm in use is very simple and easy to perform (Figure. 1).

## Conclusion

Laryngotracheobronchitis has emerged as the most common potentially life-threatening upper airway infections in children. It should be in the differential diagnosis of all children presenting with upper respiratory complaints especially if a history suggestive of witnessed aspiration and stridor can be obtained.

The primary aims of management of acute airway obstructions in children are to maintain and secure the child's airway. The second aim is establishing a diagnosis. Institutions should have algorithms to manage children with acute airway obstruction.

## List of abbreviations

BIPAP: Bi-level Positive Airway Pressure
LTB: Laryngotracheobronchitis
